# Comparison of growth performance among channel-blue hybrid catfish, ccGH transgenic channel catfish, and channel catfish in a tank culture system

**DOI:** 10.1038/s41598-021-04719-1

**Published:** 2022-01-14

**Authors:** Nermeen Y. Abass, Zhi Ye, Ahmed Alsaqufi, Rex A. Dunham

**Affiliations:** 1 School of Fisheries, Aquaculture and Aquatic Sciences, Auburn University, Auburn, AL 36849 USA; 2 Department of Agricultural Botany, Faculty of Agriculture Saba-Basha, Alexandria University, P.O. Box 21531, Alexandria, Egypt; 3 Present Address: Department of Biochemistry, University of Washington, Seattle, WA 98195 USA; 4 Present Address: Department of Aquaculture and Animal Production, College of Agriculture and Food Sciences, King Faisal University, P.O. Box 31982, Al-Ahsa, Saudi Arabia

**Keywords:** Biotechnology, Genetics

## Abstract

Fish is an essential source of high-quality protein for people worldwide. The present study was designed to compare the growth performance among the channel-blue hybrid catfish, channel catfish transgenic for the channel catfish growth hormone (ccGH) cDNA driven by the antifreeze protein promoter from an ocean pout *Zoarces americanus* (opAFP-ccGH), and non-transgenic channel catfish control. Mean body weight of channel-blue hybrid catfish was 15.80 and 24.06% larger than non-transgenic channel catfish control at 4 and 18 months of age, respectively. However, transgenic opAFP-ccGH channel catfish were 5.52 and 43.41% larger than channel-blue hybrid catfish and 22.19 and 77.91% larger than their controls at 4 and 18 months of age, respectively. Significant differences in mean body weight between the sexes within all genetic types were found. Males were larger than females (*P* < 0.001). However, mean body weight of non-transgenic males was not larger than transgenic opAFP-ccGH females or male and female hybrid catfish. Condition factor of transgenic opAFP-ccGH channel catfish was higher (*P* < 0.05) than that of full-sibling, non-transgenic channel catfish and hybrid catfish. The mean percentage body weight gain of GH transgenic channel catfish was 559%, the channel-blue hybrid catfish was 384.9% and their non-transgenic controls channel catfish was 352.6%.

## Introduction

Aquaculture is one of the fastest growing production sectors in the world^1^. Aquaculture production reached 114.5 million tons in 2018^2^. Catfish (*Ictalurus* sp.) represent the most important aquaculture sector raised for human consumption in the United States and channel-blue hybrid catfish constitutes 50–70% of catfish production^3^. Catfish production peaked at 350 million kg in 2000 then declined to 127 million kg in 2008 and currently stands at 158 million kg^4–8^. The majority of catfish production occurs in the states of Alabama, Arkansas, and Mississippi^9^. The catfish industry accounts for 95% of the United States total sales^9^. The hybrid resulting from the mating of channel catfish, *Ictalurus punctatus*, ♀ × blue catfish, *I. furcatus*, ♂ is superior to channel catfish because of its faster growth, better feed conversion, tolerance of low oxygen, increased resistance to many diseases, tolerance to harvest by seining, higher dress out and fillet yields^10–17^. However, The hybrid had negative heterosis for survival under sub-zero temperature^18^ and salinity^19^. The channel catfish ♀ × blue catfish ♂ hybrid grows faster than the reciprocal hybrid (blue catfish ♀ × channel catfish ♂)^20^.

Growth is one of the most important economic traits in aquaculture. Improvements in growth rates could reduce production costs, shorten the duration of the culture cycle needed for farm-raised fish to reach market size and increase profit. Gene transfer is an effective way to improve somatic growth and production in aquaculture^21^. Gene transfer has been used to produce various fast-growing transgenic fish species such as Atlantic salmon (*Salmo salar*)^22^, Nile tilapia (*Oreochromis niloticus*)^23^, mud loach (*Misgurnus mizolepis*)^24^, common carp (*Cyprinus carpio*)^25^, coho salmon (*Oncorhynchus kisutch*)^21^, rainbow trout (*Oncorhynchus mykiss*)^26^, and channel catfish (*Ictalurus punctatus*)^27,28^.

Growth hormone (GH) transgenesis can result in greatly increased growth rate in fish from 2- to an incredible 40-fold^27,29^, and the use of this technology for aquaculture production is now approved in the USA and Canada for triploid Atlantic salmon (*Salmo salar*) containing a chinook salmon GH transgene driven by the ocean pout antifreeze promoter (opAFP-GHc2)^30,31^. The growth rate of channel catfish, *Ictalurus punctatus*, was improved by 23–33% by transferring salmonid growth hormone gene^29,32^, and improved by 83–100% at 0.0 and 2.5 ppt salinities^27^ and 23–80% by transferring channel catfish growth hormone gene^28^.

The objective of this study was to compare the growth performance among channel catfish, *Ictalurus punctatus,* ♀ × blue catfish, *I. furcatus,* ♂ hybrids, channel catfish transgenic for the channel catfish growth hormone (ccGH) cDNA driven by the antifreeze protein promoter from an ocean pout *Zoarces americanus* (opAFP-ccGH) and their non-transgenic channel catfish control. GH transgenesis can result in different gains in growth rate in channel catfish^27,28^ and hybridization has resulted in heterosis for growth rate in the channel-blue hybrid catfish^12^, but comparison of growth performance between channel-blue hybrid and the GH transgenesis has not been examined.

## Results

There was a significant difference between mean body weights of transgenic channel catfish and their non-transgenic siblings at 4 months of age (*P* < 0.05). However, there were no significant differences between mean body weight of hybrid catfish and non-transgenic channel catfish and no significant differences between mean body weight of hybrid catfish and transgenic channel catfish (Table 1). No significant differences were observed among mean body length of transgenic channel catfish, hybrid catfish and their non-transgenic siblings at 4 months of age (*P* > 0.05) (Table 2).

**Table 1:** Mean body weight (BW, g) ± standard deviation (SD) and coefficient of variation (CV), and range of BW at 4 and 18 months of age among the F_1_ generation of channel catfish, *Ictalurus punctatus*, ♀ × blue catfish, *I. furcatus*, ♂ hybrid catfish, transgenic channel catfish (*Ictalurus punctatus*) containing channel catfish growth hormone (ccGH) cDNA driven by the ocean pout *Zoarces americanus* antifreeze protein promoter (opAFP-ccGH), and full-sibling control channel catfish.

Genotype^A^	Initial body weight (g)				Final body weight (g)			
	*N*	*F*	BW (g) ± SD (CV)	Range	*N*	*F*	BW (g) ± SD (CV)	Range
Hybrid	60	4.06*	8.87 ± 2.62^ab^ (0.30)	5.5–14.0	141	12.98**	43.01 ± 17.42^b^ (0.41)	13.0–90.0
opAFP-ccGH (T)	14		9.36 ± 2.85^a^ (0.30)	5.0–14.0	14		61.68 ± 26.56^a^ (0.43)	30.5–122.5
opAFP-ccGH (N)	56		7.66 ± 1.69^b^ (0.22)	4.0–11.0	56		34.67 ± 17.60^c^ (0.51)	12.5–88.0

Fish were reared in a 100-L tank at 500 fish/tank.

Within a column, means that do not differ at *P* = 0.05 are followed by the same superscript (Duncan’s multiple range test) among different genetic groups. *p < 0.05 *or* **p ≤ 0.0001.

^A^Hybrid = the channel catfish, *Ictalurus punctatus*, ♀ × blue catfish, *I. furcatus*, ♂ hybrid catfish, opAFP-ccGH (T) = channel catfish transgenic for channel catfish growth hormone (ccGH) cDNA driven by the ocean pout *Zoarces americanus* antifreeze protein promoter, opAFP-ccGH (N) = channel catfish non-transgenic for channel catfish growth hormone (ccGH) cDNA driven by the ocean pout *Zoarces americanus* antifreeze protein promoter.

**Table 2:** Mean body length (BL, cm) ± standard deviation (SD) and coefficient of variation (CV), and range of BL at 4 and 18 months of age among the F_1_ generation of channel catfish, *Ictalurus punctatus*, ♀ × blue catfish, *I. furcatus*, ♂ hybrid catfish, transgenic channel catfish (*Ictalurus punctatus*) containing channel catfish growth hormone (ccGH) cDNA driven by the ocean pout *Zoarces americanus* antifreeze protein promoter (opAFP-ccGH), and full-sibling control channel catfish.

Genotype^A^	Initial body length (cm)				Final body length (cm)			
	*N*	*F*	BL (cm) ± SD (CV)	Range	*N*	*F*	BL (cm) ± SD (CV)	Range
Hybrid	60	3.84	10.48 ± 0.93^a^ (0.09)	8.0–12.0	141	5.10 *	17.46 ± 2.74^b^ (0.16)	13.0–36.0
opAFP-ccGH (T)	14		10.41 ± 1.60^a^ (0.09)	6.0–12.5	14		19.00 ± 3.74^a^ (0.20)	13.0–25.0
opAFP-ccGH (N)	56		9.91 ± 0.85^a^ (0.15)	8.0–11.5	56		16.38 ± 2.37^b^ (0.14)	12.0–23.0

Fish were reared in a 100-L tank at 500 fish/tank.

Within a column, means that do not differ at *P* = 0.05 are followed by the same superscript (Duncan’s multiple range test) among different genetic groups. *p < 0.05.

^A^Hybrid = the channel catfish, *Ictalurus punctatus*, ♀ × blue catfish, *I. furcatus*, ♂ hybrid catfish, opAFP-ccGH (T) = channel catfish transgenic for channel catfish growth hormone (ccGH) cDNA driven by the ocean pout *Zoarces americanus* antifreeze protein promoter, opAFP-ccGH (N) = channel catfish non-transgenic for channel catfish growth hormone (ccGH) cDNA driven by the ocean pout *Zoarces americanus* antifreeze protein promoter.

**Figure 1:**
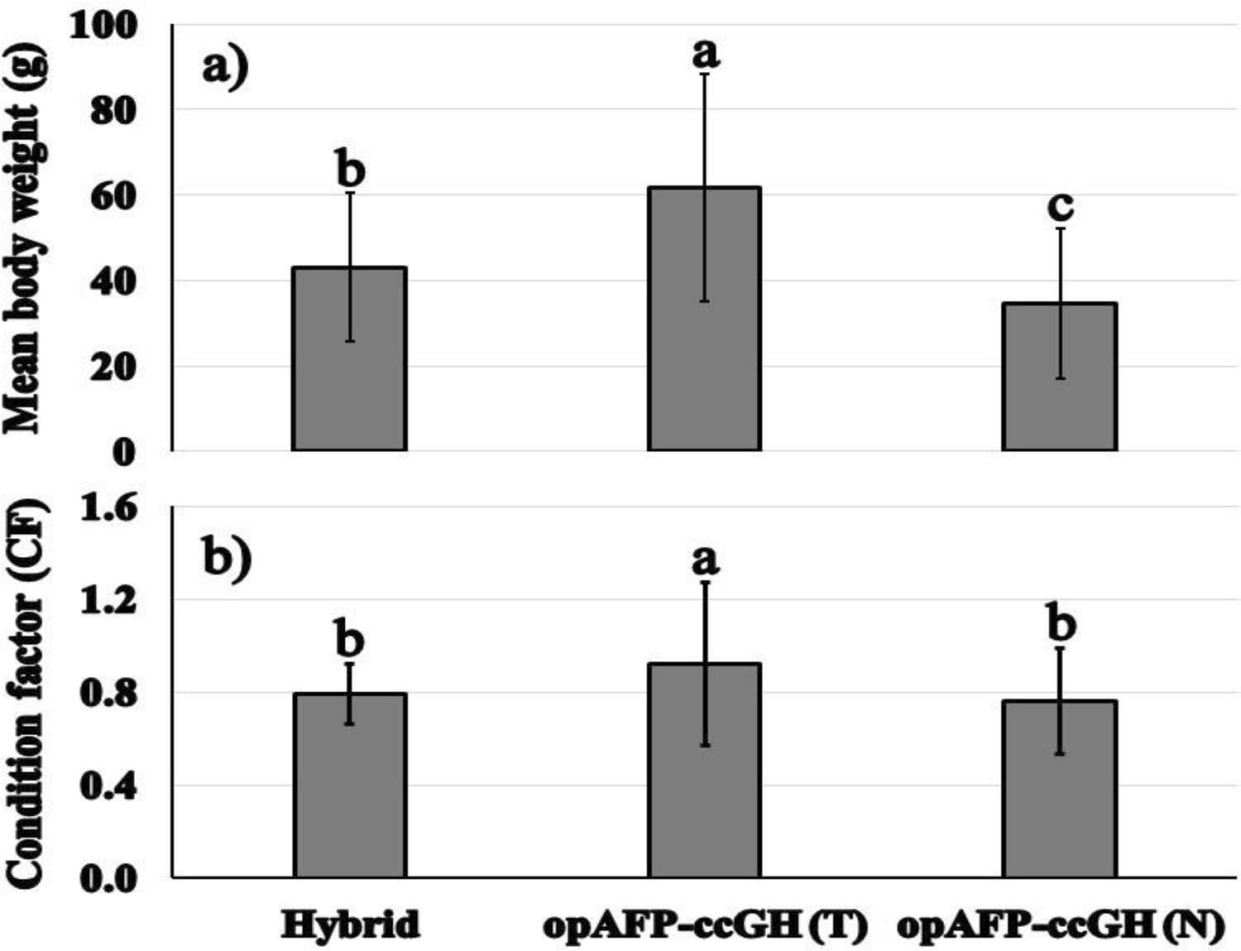
Comparison of different genetic groups of the F_1_ generation of hybrid catfish, transgenic channel catfish containing channel catfish growth hormone (ccGH) cDNA driven by the ocean pout *Zoarces americanus* antifreeze protein promoter, and channel catfish. (**a**) mean final body weight (g) ± SD, and (**b**) condition factor ± SD. Fish were 18 months of age. Fish were reared in a 100-L tank at 500 fish/tank. Treatments, Hybrid = the channel catfish, *Ictalurus punctatus*, ♀ × blue catfish, *I. furcatus*, ♂ hybrid catfish, opAFP-ccGH (T) = channel catfish transgenic for channel catfish growth hormone (ccGH) cDNA driven by the ocean pout antifreeze protein promoter, opAFP-ccGH (N) = channel catfish non-transgenic for channel catfish growth hormone (ccGH) cDNA driven by the ocean pout antifreeze protein promoter. Means that do not differ at *P* = 0.05 are followed by the same superscript (Duncan’s multiple range test) among different genetic groups (*P* < 0.0001).

At 18 months of age in tanks, the largest transgenic opAFP-ccGH was 3.5 times that of the average non-transgenic and 2.8 times that of the hybrid catfish. The average body weight of hybrid catfish was 43.01±17.42 g, transgenic opAFP-ccGH channel catfish was 61.68±26.56 g, and non-transgenic channel catfish was 34.67±17.60 g (*F* = 12.98; *P* < 0.0001) (Table 1 and Fig. 1a). However, the average total length of hybrid catfish was 17.46±2.74 cm, transgenic opAFP-ccGH channel catfish was 19.00±3.74 cm, and non-transgenic channel catfish was 16.38±2.37 cm (*F* = 5.10; *P* < 0.05) (Table 2). Transgenic opAFP-ccGH channel catfish grew 1.8- fold larger than their non-transgenic full-siblings, and 1.4- fold larger than hybrid catfish (*P* < 0.0001). The hybrid catfish grew 1.3- fold larger than non-transgenic channel catfish (Table 1 and Fig. 1a), and the largest transgenic opAFP-ccGH channel catfish was larger than the largest control (non-transgenic) channel catfish and hybrid catfish. The transgenic fish were larger than controls at 18 months, and their rate of growth was even more rapid than controls from 4 to 18 months, as the mean percentage body weight gain of GH transgenic channel catfish was 559%, the channel-blue hybrid catfish was 384.9% and their non-transgenic channel catfish control was 352.6%.

At 18 months of age in tanks, condition factor (CF) of hybrid catfish was 0.79±0.13, transgenic opAFP-ccGH was 0.92±0.35, and non-transgenic was 0.76±0.23. Condition factor (CF) of non-transgenic channel catfish was similar to hybrid catfish (*P* = 0.54) (Fig. 1b). However, there was a significant difference between transgenic opAFP-ccGH channel catfish and the non-transgenic channel catfish and hybrid catfish (Fig. 1b).

**Figure 2:**
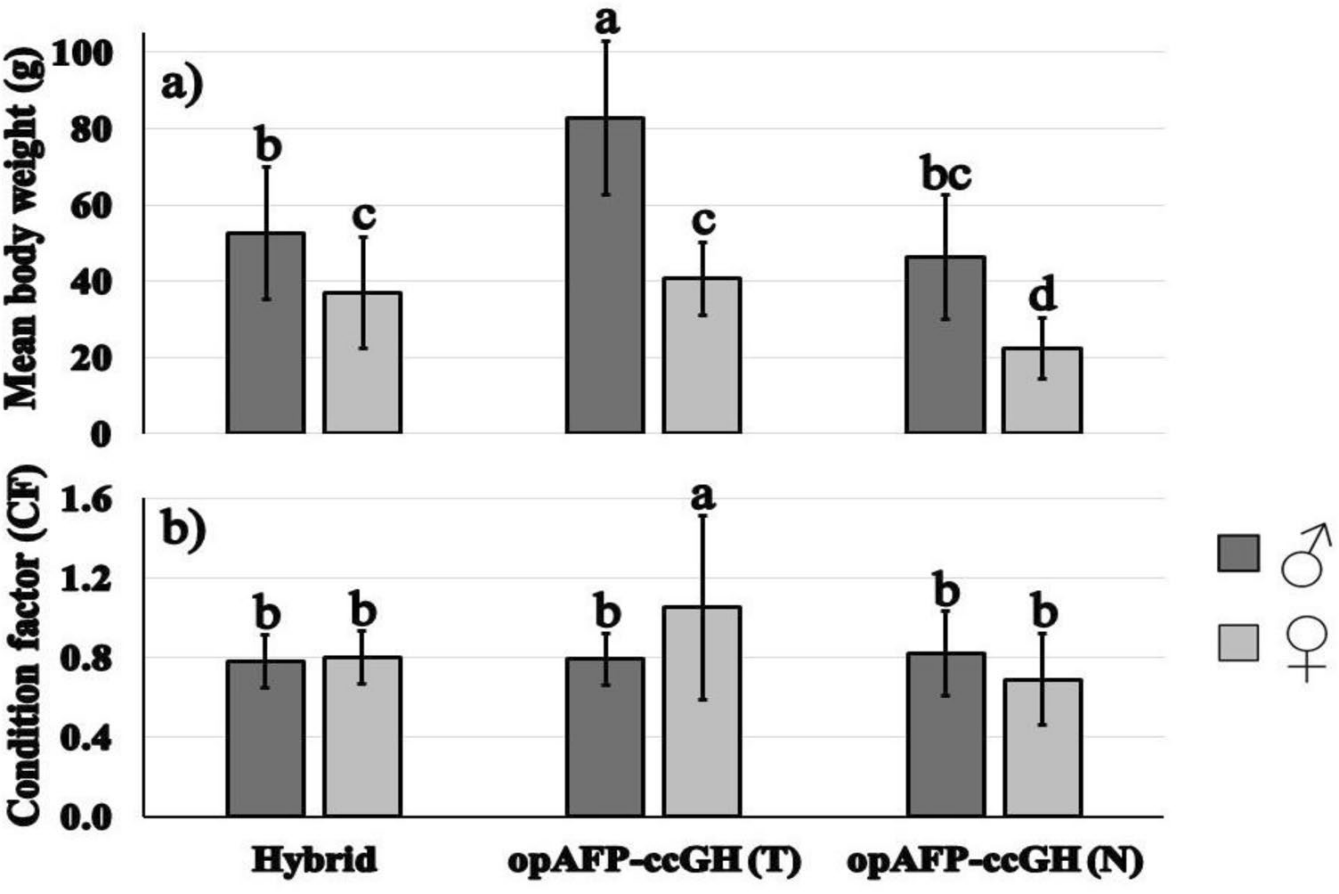
Comparison of male and female F_1_ channel catfish, *Ictalurus punctatus*, ♀ × blue catfish, *I. furcatus*, ♂ hybrid catfish, transgenic channel catfish containing channel catfish growth hormone (ccGH) cDNA driven by the ocean pout *Zoarces americanus* antifreeze protein promoter, and full-sibling control channel catfish. (**a**) mean final body weight (g) ± SD, and (**b**) condition factor ± SD. Fish were 18 months of age. Fish were reared in a 100-L tank at 500 fish/tank. Treatments, Hybrid = the channel catfish, *Ictalurus punctatus*, ♀ × blue catfish, *I. furcatus*, ♂ hybrid catfish, opAFP-ccGH (T) = channel catfish transgenic for channel catfish growth hormone (ccGH) cDNA driven by the ocean pout antifreeze protein promoter, opAFP-ccGH (N) = channel catfish non-transgenic for channel catfish growth hormone (ccGH) cDNA driven by the ocean pout antifreeze protein promoter. Means that do not differ at *P* = 0.05 are followed by the same superscript (Duncan’s multiple range test).

The average body weight of 18 months old hybrid catfish males, hybrid catfish females, transgenic opAFP-ccGH males, transgenic opAFP-ccGH females, non-transgenic opAFP-ccGH males, and non-transgenic opAFP-ccGH females was 52.58±17.37 g, 36.89±14.53 g, 82.17±22.01 g, 41.58±9.99 g, 45.22±16.24 g, and 22.09±7.86 g (*F* = 36.14; *P* < 0.0001) (Table 3 and Fig. 2a), respectively. The average total length of 18 months old hybrid catfish males, hybrid catfish females, transgenic opAFP-ccGH males, transgenic opAFP-ccGH females, non-transgenic opAFP-ccGH males, and non-transgenic opAFP-ccGH females was 18.98±3.02 cm, 16.48±2.02 cm, 21.50±1.87 cm, 16.67±2.56 cm, 17.59±1.97 cm, and 15.36±2.93 cm (*F* = 22.21; *P* < 0.0001) (Table 3), respectively.

**Table 3:** Mean body weight (g), mean body length (cm) ± standard deviation (SD), coefficient of variation (CV), and range for the male and female F_1_ channel catfish, *Ictalurus punctatus*, ♀ × blue catfish, *I. furcatus*, ♂ hybrid catfish, transgenic channel catfish (*Ictalurus punctatus*) containing channel catfish growth hormone (ccGH) cDNA driven by the ocean pout *Zoarces americanus* antifreeze protein promoter (opAFP-ccGH), and full-sibling control channel catfish. Fish were 18 months of age.

Genotype^A^	Sex	*N*	*F* for mean body weight	Body weight (g)		*F* for mean body length	Body length (cm)	
				Mean ± SD (CV)	Range		Mean ± SD (CV)	Range
Hybrid	Males	55	36.14**	52.58 ± 17.37^b^ (0.33)	17.0–90.0	22.21**	18.98 ± 3.02^b^ (0.16)	14.0–36.0
opAFP-ccGH (T)		7		82.17 ± 22.01^a^ (0.27)	61.0–122.5		21.50 ± 1.87^a^ (0.09)	21.0–25.0
opAFP-ccGH (N)		29		45.22 ± 16.24^bc^ (0.36)	23.0–88.0		17.59 ± 1.97^bc^ (0.11)	14.0–23.0
Hybrid	Females	86		36.89 ± 14.53^c^ (0.39)	13.0–85.5		16.48 ± 2.02^ cd^ (0.12)	13.0–22.0
opAFP-ccGH (T)		7		41.58 ± 9.99^c^ (0.24)	30.5–57.0		16.67 ± 2.56^ cd^ (0.15)	13.0–20.0
opAFP-ccGH (N)		27		22.09 ± 7.86^d^ (0.36)	12.5–43.0		15.36 ± 2.93^d^ (0.19)	12.0–20.0

Fish were reared in a 100-L tank at 500 fish/tank.

Within a column, means that do not differ at *P* = 0.05 are followed by the same superscript (Duncan’s multiple range test) among the male and female of different genetic groups. **p ≤ 0.0001.

^A^Hybrid = the channel catfish, *Ictalurus punctatus,* ♀ × blue catfish, *I. furcatus*, ♂ hybrid catfish, opAFP-ccGH (T) = channel catfish transgenic for channel catfish growth hormone (ccGH) cDNA driven by the ocean pout *Zoarces americanus* antifreeze protein promoter, opAFP-ccGH (N) = channel catfish non-transgenic for channel catfish growth hormone (ccGH) cDNA driven by the ocean pout *Zoarces americanus* antifreeze protein promoter.

Significant differences in body weight were observed between the sexes. Body weight of the males was significantly heavier compared with those of the females (*P* < 0.001) of different genetic groups. However, there were no significant differences among mean body weight of male non-transgenic channel catfish, female transgenic GH channel catfish and female hybrid catfish, and no significant differences between mean body weight of the male non-transgenic channel catfish and male hybrid catfish (Table 3 and Fig. 2a).

The body weight of 18-month-old transgenic opAFP-ccGH channel catfish males was 1.98 times higher than that of their females and 1.56 times higher than hybrid catfish males. However, the body weight of hybrid catfish males was 1.43 times higher than that of their females and 1.16 times higher than non-transgenic channel catfish males. The body weight of non-transgenic channel catfish males was 2.05 times higher than that of their corresponding females (*P* < 0.0001) (Table 3 and Fig. 2a).

The total length of 18-months-old hybrid catfish males was 1.15 times longer than that of their females and 1.08 times longer than non-transgenic males. However, transgenic opAFP-ccGH males were 1.29 times longer than that of their females and 1.13 times longer than CB hybrid catfish males. The body length of non-transgenic males was 1.15 times longer than that of their females (*P* < 0.0001) (Table 3).

The condition factor (CF) of 18- months- old hybrid catfish males, hybrid catfish females, transgenic opAFP-ccGH males, transgenic opAFP-ccGH females, non-transgenic opAFP-ccGH males, and non-transgenic opAFP-ccGH females was 0.78±0.13, 0.80±0.13, 0.79±0.13, 1.05±0.46, 0.82±0.21, and 0.69±0.22 (*P* = 0.0003) (Fig. 2b), respectively. Significant differences in CF were observed between GH transgenic females and all other genotypes (Fig. 2b).

Sexual development was more pronounced for the GH siblings than the non-transgenic siblings. Additionally, the skin of the non-transgenic siblings was darker than that of GH transgenic full-siblings.

## Discussion

The catfish industry has been severely challenged in recent years as a result of higher operating costs, disease problems, and competition from cheaper imported frozen fillet products from Vietnam and China^8^. A potential future tool to solve this problem is the utilization of genetically engineered catfish, which can increase yield, disease resistance, and survivability in extreme environments. In the current study, we transferred channel catfish *Ictalurus punctatus* growth hormone cDNA construct driven by the antifreeze protein promoter from an ocean pout *Zoarces americanus* (opAFP-ccGH) to channel catfish to produce ccGH transgenic channel catfish and compared the growth rate among hybrid catfish, transgenic opAFP-ccGH channel catfish, and non-transgenic channel catfish.

Transgenic opAFP-ccGH channel catfish grew 1.8- fold larger than their non-transgenic, and 1.4- fold larger than hybrid catfish. However, the hybrid catfish grew 1.3- fold larger than non-transgenic channel catfish. Our results and those of Abass et al.^27,28^ indicated that the channel catfish growth hormone (ccGH) gene was incorporated and affected their growth performance compared to their non-transgenic. This is especially significant as hybrid catfish usually grow faster than channel catfish in high density raceways^33,34^, but in the current experiment GH transgenic channel catfish grew faster than high performance hybrid catfish. The hybrids grew faster than controls as expected.

Several fish species transgenic for GH show significantly increased somatic growth^35,36^, via muscle hypertrophy or hyperplasia. Muscle mass generated in 7-month-old GH-transgenic zebrafish is due to hypertrophy^37^, but hyperplasic growth is shown in 2-month-old GH-transgenic coho salmon^38^. Channel catfish transgenic for Rous Sarcoma Virus Long Terminal Repeat (RSV-LTR) promoter-rainbow trout GH cDNA had greater numbers of mitochondria and glycogen globules, fewer fat globules, greater numbers of muscle fibers, but the same size of muscle fibers as control catfish^39^, which was virtually the same as the results of Hill et al.^38^ for GH transgenic coho salmon. In the current study, this data was not collected. However, based on the previous studies on GH channel catfish and coho salmon, it is likely that the GH catfish in the current study grew faster due to hyperplasia rather than hypertrophy of muscle fibers. Again, the zebrafish model, is not always predictive as the opposite results, muscle fiber hypertrophy, were found for GH zebrafish^37^.

GH transgenic fish also have exhibited increased rates of protein synthesis, numbers of mitochondria in the cell, lipid mobilization, numbers of glycogen globules, feeding behavior, feed conversion efficiency, metabolic rate, changes in head and body morphometrics, osmoregulation, cold and salinity tolerance, and age-at-maturity^18,27,40–43^. Growth hormone transgenic Atlantic salmon had decreased feed consumption of 25% and time to reach market weight by 40% compared to non-transgenic fish^43^.

F_1_ and F_2_ transgenic common carp *Cyprinus carpio* expressing rainbow trout cDNA grew 3% to 37% and 0% to 49% faster than their non-transgenic, respectively, depending upon family^25^. One year old, transgenic Atlantic salmon *Salmo salar *expressing a chinook salmon GH cDNA driven by the ocean pout antifreeze protein gene from an ocean pout grew 2- to 6- fold larger than the non-transgenic control, and the largest transgenic fish was 13 times larger than the average non-transgenic^22^. Transgenic tilapia *Oreochromis niloticus* expressing a chinook salmon GH cDNA driven by the ocean pout antifreeze protein grew 2.5- to 4- fold faster than non-transgenic controls^23,44–46^. At 16 weeks old, transgenic channel catfish expressing channel catfish GH (ccGH) cDNA driven by the opAFP grew 1.4- to 1.6- fold larger than their non-transgenic full-siblings, and transgenic channel catfish expressing ccGH cDNA driven by the rtMT grew 1.23- to 1.8- fold larger than their non-transgenic full-siblings (*P* < 0.0001)^28^. The growth rate of the F_1_ fingerling transgenic channel catfish expressing coho salmon GH cDNA driven by the RSV-LTR promoter was improved by 23–26%^32^. GH plays an important and critical role in regulating mRNA expression for growth-related genes^47^. The response to GH transgene expression has been variable in fish perhaps due to differences in the promoter, construct, genetic background, environment, stocking density or length of study.

In the current study, hybrid catfish had higher growth rate than non-transgenic channel catfish. Individual heterosis (dominance effects) had a strong positive effect on growth rates for channel catfish female, *Ictalurus punctatus*, × blue catfish, *I. furcatus*, male hybrid catfish^15^. Previous research has also shown higher growth rate for hybrid catfish than channel catfish^19^. However, Dunham et al.^12^found that the channel catfish had more rapid growth than hybrid catfish in cages, but slower than hybrid catfish in ponds (*P* < 0.01) and Small^48^ demonstrated that growth of hybrid catfish is lower than channel catfish when they are reared in small tank/aquaria systems. Differences in body weight were also found among hybrids produced from different parental strains or families^11^. Dunham et al.^11^ reported that the Marion Kansas (MK) channel catfish and Kansas Select (KS) channel catfish grew faster to 100 g than hybrid catfish during the first season in low density ponds. However, the hybrid catfish were slightly larger 1.3% than KS channel catfish but did not differ in body weight from MK channel catfish at the end of the second season in earthen ponds (the dam of the hybrid was not KS or MK). The F_1_hybrid catfish grew15–20% faster than the channel catfish^11,15^. Weight gain for KR channel catfish ♀ × D&B blue catfish ♂ hybrid catfish was bet ter, 94% he t eros is, than KR channel catfish in tank systems at 0 ppt sodium chloride^19^.

In the present study, significant differences in body weight were found between the sexes. Body weight of the males was significantly higher compared with those of the females of different genetic groups. Sexual dimorphism in growth has been observed in several fish species of commercial interest^49^. Channel catfish males grew faster than females when stocked together in ponds^50–53^ due to the genetic component^54^.

The response to growth hormone transgene insertion in channel catfish is affected by construct, age/size and sex. F_1_ transgenic opAFP-ccGH and rainbow trout metallothionein promoter-channel catfish growth hormone transgene (rtMT-ccGH) channel catfish grew 90% faster than controls^27^. In a second experiment, opAFP-ccGH and rtMT-ccGH transgenic channel catfish fry grew 50–58% larger than their non-transgenic full-siblings^28^. For large fingerling channel catfish harboring RSV-LTR promoter- salmonid growth hormone transgenes growth was improved by 0–33%^32^.

For food size channel catfish fish with the opAFP-ccGH and rtMT-ccGH constructs, the result s were more dramatic with body weight increases of 165–227%^55^. Sexually dimorphic responses to GH transgenic channel catfish were the opposite after sexual maturation^55^. At 16 months of age, the transgenic ccGH males had slightly higher body weight than the transgenic ccGH females . However, at 48 months of age, the body weight of the transgenic ccGH females was slightly higher than that of the transgenic GH males. Males were larger than females in non-transgenic ccGH siblings at both 16 and 48 months of age^55^. Similarly, strong sexually dimorphic growth between female and male was observed in the 5750A transgenic coho salmon strain with the females being larger, but was not in M77 strain harbouring the same GH gene construct^56^.

In the current study, h ybrid catfish males, transgenic GH channel catfish males, and non-transgenic channel catfish males were 42.53%, 97.62%, and 104.71% heavier and 15.17%, 28.97%, and 14.52% longer than hybrid catfish females, transgenic GH channel catfish females, and non-transgenic channel catfish females, respectively at 18 months of age. Simco et al.^54^ reported that at 10 mont hs old sex did not influence the growth rates of channel catfish weighing less than 50 g. However, at 26 months old, mal e channel catfish were 10% longer and 37% heavier than females. Bondari et al.^57^ reported that male channel catfish were 7.4% longer and 22.4% heavier than female channel catfish for fish averaging 500–600 g. Brooks et al.^58^ and Dunham et al.^50^ found that male channel catfish were 40% and 29% heavier, respectively, than females. Sex had an increasing influence on growth rate with increasing size and age in channel catfish^54^. Dunham et al.^59^ observed large differences between weight and sex among F_1_ channel × blue hybrids produced from different strains, and other strain crosses of F_1_ hybrids showed no difference between sex and weight. Heterosis of interspecific hybrids can vary depending upon the species, strain, genetics, life stage, age, stocking density, feeding regium, environmental factors, and genotype-environment interactions.

The GH transgenic channel catfish had greater sexual development than non-transgenic full-siblings based on head development and genital development. This is complex and is likely not a direct effect of the GH transgene, but likely due to the transgenic individuals reaching a size that initial sexual maturation effects are emerging.

There was a significant difference in the condition factor (CF) among hybrid catfish, GH transgenic channel catfish, and their non-transgenic siblings. However, there was no difference between non-transgenic channel catfish and hybrid catfish for CF. The CF was less than one for all genotypes. Our results demonstrated that the GH transgenic channel catfish had higher CF than their controls. The same result was obtained for the GH transgenic common carp, which had higher CF than their controls^60^.

In contrast, there was no difference in the CF of the GH transgenic channel catfish and their full-sibling control fry evaluated by Abass et al.^27^ and fingerlings evaluated by Abass et al.^28^ except for one opAFP-ccGH  family^28^. Leggatt et al.^61^ reported that the transgenic coho salmon had greater CF than their controls, although this difference was not present at all time points in all strains. No differences in CF between the sexes were found for hybrid catfish and non-transgenic channel catfish. However, the CF of ccGH cDNA transgenic channel catfish females was higher than that of all other male and female genotypes as apparently their relative weight was increasing more rapidly than their relative length compared to their controls and hybrid catfish.

In conclusions, our results demonstrated that the transgenic channel catfish possessing channel catfish growth hormone cDNA grew faster than hybrid catfish in a high-density tank environment. However, more research is needed u nder pond culture to compare the growth performance among transgenic hybrid catfish, normal hybrid catfish, and GH transgenic channel catfish as hybrid catfish is currently the superior genotype for pond culture^62^.

## Materia ls and methods

All experimental protocols used in this experiment were approved by the Auburn University Institutional Animal Care and Use Committee (AU-IACUC) before the experiment was initiated, and followed Association for Assessment and Accreditation of Laboratory Animal Care (AAALAC) protocols and guidelines. The study was carried out in compliance with the ARRIVE guidelines.

### Broodstock and fry production

The P_1_ transgenic and control broodstock used for this study were from the Catfish Genetics Research Unit, School of Fisheries Aquaculture and Aquatic Sciences, Auburn University, AL, USA. The P_1_ transgenic fish were produced via electroporation^63,64^. Broodstock were tested to determine if they were transgenic prior to spawning^18,27^. Three F_1_ (P_1_ wild-type Kansas random (KR)^65^ females mated with three KR transgenic males) families of channel catfish, *Ictalurus punctatus*, transgenic for the channel catfish growth hormone (ccGH) cDNA driven by the ocean pout *Zoarces americanus* antifreeze protein promoter (opAFP-ccGH)^18,27^ were induced to spawn by injection with luteinizing hormone releasing hormone analogue (LHRHa) implants at 85 µg/kg female body weight^66^. D&B (DB)^65^ blue catfish males were used to fertilize Kansas random (KR) channel catfish females to produce three hybrid catfish families. The stripped eggs were fertilized artificially with sperm from the males.

### F_1_ fish culture

When egg masses were obtained, they were placed in wire mesh baskets in hatching troughs with constant water flow and aeration. Calcium chloride solution was continually dripped into the trough to ensure 40–50 ppm hardness. Eggs were gently agitated with a paddlewheel beginning 2 h after spawn collection. The egg masses were prophylactically treated with 100 ppm formalin or 32 ppm copper sulfate every 8 h to avoid fungus^67^. The treatments were terminated 12 h before hatch. The pH ranged from 7 to 7.3 and DO from 6.8 to 7.8 mg/L. Water flow through each tank was maintained at least 15 L/min to ensure an exchange rate of at least twice per hour.

Catfish embryos began hatching in 7 days with a water temperature between 26–28 °C. Transgenic and non-transgenic channel catfish were in the same tank/environment from the moment of fertilization since they were full-siblings. They consumed their yolk sac and began to swim up stage 3 days post hatching. Larvae were first fed *Artemia nauplii* (San Francisco Bay Brand, Inc. Newark, CA), three times a day and stocked into flow-through tanks with densities of 1000 fish per 90 L tank, then fry were fed Aquamax® Fry Powder (Cat#: 000-7684, Purina Mills, St. Louis, MO) three times daily. Hybrid catfish was in one tank and mixed transgenic and non-transgenic channel catfish were in another tank. After 8 weeks, they were fed Aquamax® Fingerling Starter 200 twice a day to satiation (Cat#: 000-5554, Purina Mills, St. Louis, MO). After 4 months, fingerling swere mixed and moved to a recirculating system at a stocking density of 500 (250 fingerlings from hybrid catfish and 250 fingerlings from channel catfish) fingerlings per 100 L tank. Sample body weights were taken at this time, and the sample of channel catfish was genotyped for the transgene. Hybrid catfish (mean weight: 8.87 ± 2.62 g; mean length: 10.48 ± 0.93 cm), transgenic opAFP-ccGH (mean weight: 9.36 ± 2.85 g; mean length: 10.41 ± 1.60 cm), and non-transgenic opAFP-ccGH (mean weight: 7.66 ± 1.69 g; mean length: 9.91 ± 0.85 cm) were used in this phase of the experiment. Hybrid catfish and transgenic channel catfish were not different in size at this timepoint. Fingerlings were fed Aquamax® Fingerling Starter 300 twice a day to satiation (Cat#: 000-5555, Purina Mills, St. Louis, MO). After 18 months, 211 juven ile fish were randomly selected; body weight and length of each fish were recorded. Anal fin tissues (200 mg) from 70 channel catfish were collected at 4 and 18 months of age. These sam ples were immediately stored at −80 °C until DNA extrac tio n .

### Growth parameters

Growth response parameters were calculated as follows:

$$\mathrm{Weight \,\,gain }\,\,(\mathrm{\%}) =100\times \frac{\mathrm{FBW }-\mathrm{ IBW}}{\mathrm{IBW}}$$

where FBW and IBW = final and initial body weight (g), respectively.

$$\mathrm{Condition\,\, factor }\,\,(\mathrm{CF})=\frac{\begin{array}{c}\\ 100\times W\end{array}}{\begin{array}{c}\\ {\mathrm{L}}^{3}\end{array}}$$

where W is weight (g) and L is total length (cm).

No mortality was recorded for each genotype before or during the experiment.

### Morphological examination

**Figure 3:**
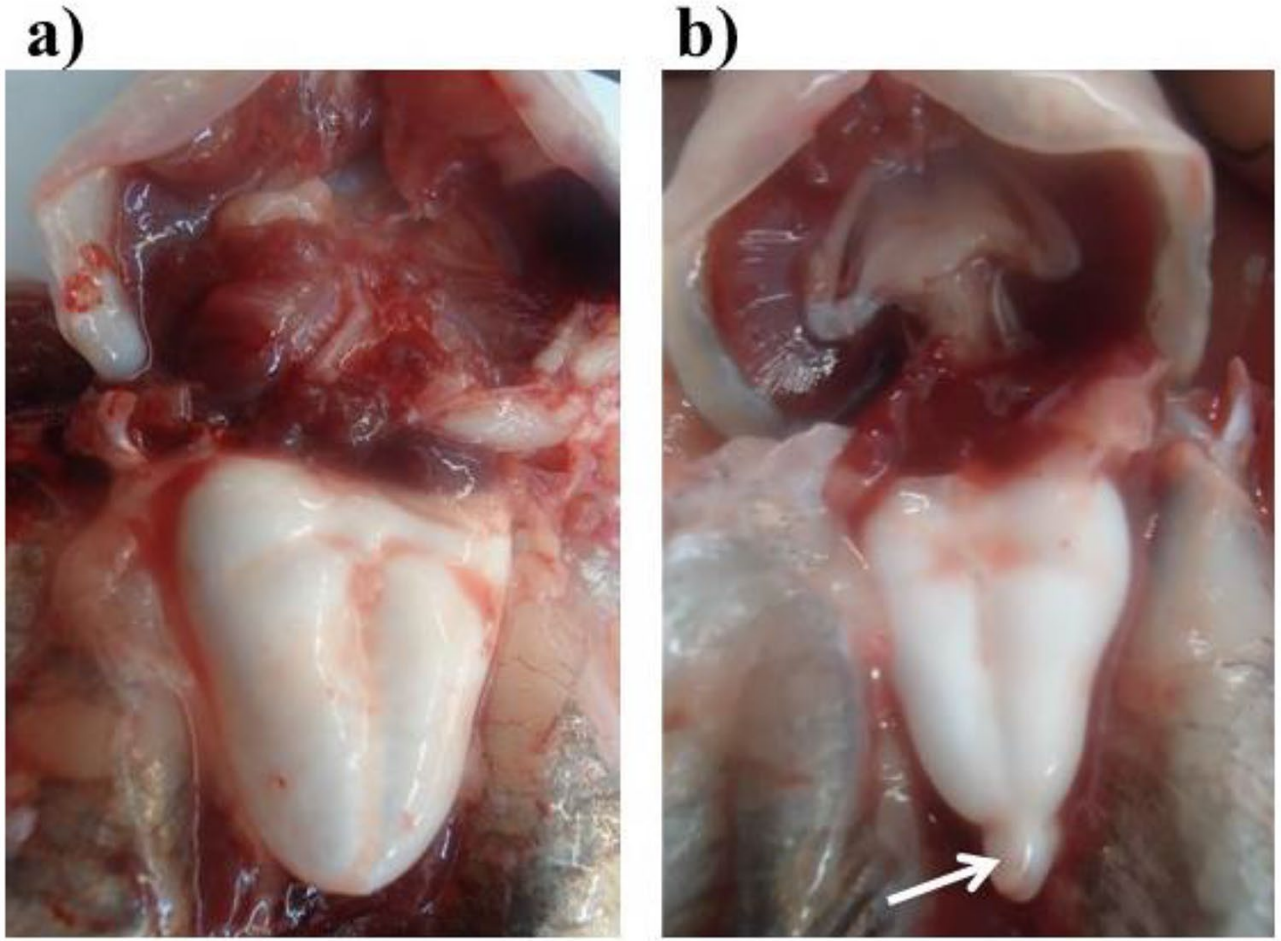
Kansas random (KR) swim bladder of a channel catfish, *Ictalurus punctatus*, (**a**) compared with that of a similar-sized channel catfish ♀ × blue catfish, *I. furcatus*, ♂ hybrid catfish (**b**). Channel catfish have a single lobed, heart shaped swim bladder. The channel catfish ♀ × blue catfish ♂ hybrid catfish has a bi-lobed swim bladder, but the first anterior chamber has a large, heart-shaped lobe while the second posterior lobe is a small, attached protrusion. (Photographs by Nermeen Y. Abass).

Each fish was compared to known channel catfish and channel catfish ♀ × blue catfish ♂ hybrids of the same size for body shape, color, and swim bladders (Fig. 3)^20^. Fish were sexed by examination of the urogenital area.

### Transgene identification

Genomic DNA was extracted using proteinase K digestion followed by protein precipitation and DNA ethanol precipitation as described in the protocol of Kurita et al.^68^ with some modifications. The quality and quantity of DNA samples were confirmed using ethidium bromide-stained agarose gels and NanoDrop 2000|2000c Spectrophotometers. All extracted samples had an A260/280 ratio greater than 1.8 and were diluted to 200 ng.

**Figure 4:**
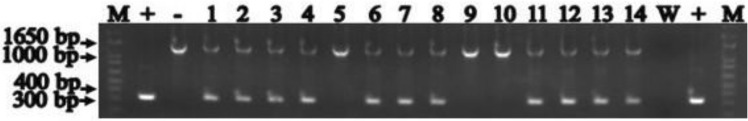
Example of PCR analyses of the F_1_ KR generation of transgenic and non-transgenic (full-sibling) channel catfish, *Ictalurus punctatus*, total DNA. Analysis of channel catfish growth hormone (ccGH) cDNA; 332 bp. 1:14 = different DNA samples from the F_1_ KR generation of transgenic and non-transgenic (full-sibling) containing channel catfish growth hormone (ccGH) cDNA driven by the ocean pout *Zoarces americanus* antifreeze protein promoter (18 months); +  = positive control, plasmid DNA; − = negative control, DNA sample from non-transgenic (wild-type) control fish;  W  =  water control, PCR reaction of water served as negative control; and marker lanes contain 1 Kb plus DNA ladders (Invitrogen). This figure was cropped, and the full-length gel is presented in Supplementary Fig. S1. PCR products visualized with a Molecular Imager Gel Doc XR + System using Image Lab Software (Bio-Rad Laboratories, Inc, Hercules, CA)^69^. (Photograph by Nermeen Y. Abass).

Transgenic channel catfish samples were screened by PCR with specific primers as described in Abass et al.^18,27,28^ with some modifications. PCR products were subject to electrophoresis on an ethidium bromide 1.2% TAE agarose gel and visualized with a Molecular Imager Gel Doc XR+ System using Image Lab Software (Bio-Rad Laboratories, Inc, Hercules, CA)^69^. PCR products were observed of the expected size of 332 bp for the channel catfish *Ictalurus punctatus* growth hormone (ccGH) cDNA (Fig. 4).

### Statistical analysis

Body weight, total body length, and condition factor (CF) were expressed as mean ± SD, and subjected to one-way ANOVA, and significant differences among different genetics groups were assessed using Duncan’s^70^ multiple comparison test at *P* < 0.05. Statistical analyses were conducted using SAS software^71^.

## Supplementary Information

Supplementary Figure S1.
