# Supplementary material for: Comparison of growth performance among channel-blue hybrid catfish, ccGH transgenic channel catfish, and channel catfish in a tank culture system
Source: Sci Rep. 2022 Jan 14;12:740. doi: 10.1038/s41598-021-04719-1 (PMC8760261; doi:10.1038/s41598-021-04719-1)
Supplement: Supplementary file 1 — Supplementary Figure S1. [file 41598_2021_4719_MOESM1_ESM.docx]

**Supplementary Information**

**Comparison of growth performance among channel-blue hybrid catfish, ccGH transgenic channel catfish, and channel catfish in a tank culture system**

Nermeen Y. Abass^a,b*^, Zhi Ye^1,a^, Ahmed Alsaqufi^2,a^ and Rex A. Dunham^a^

^a^ School of Fisheries, Aquaculture and Aquatic Sciences, Auburn University, AL 36849, USA

^b^ Department of Agricultural Botany, Faculty of Agriculture Saba-Basha, Alexandria University, Alexandria City, P.O. Box 21531, Egypt

^1^Current address: Department of Biochemistry, University of Washington, Seattle, WA 98195, USA

^2^Current address: Department of Aquaculture and Animal Production, College of Agriculture and Food Sciences, King Faisal University, Al-Ahsa City, P.O. Box 31982, Saudi Arabia

*Corresponding author: Tel.: + 2 03 5831646; fax: + 2 035832008.

E-mail address: [n.y.abass@alexu.edu.eg](mailto:n.y.abass@alexu.edu.eg)


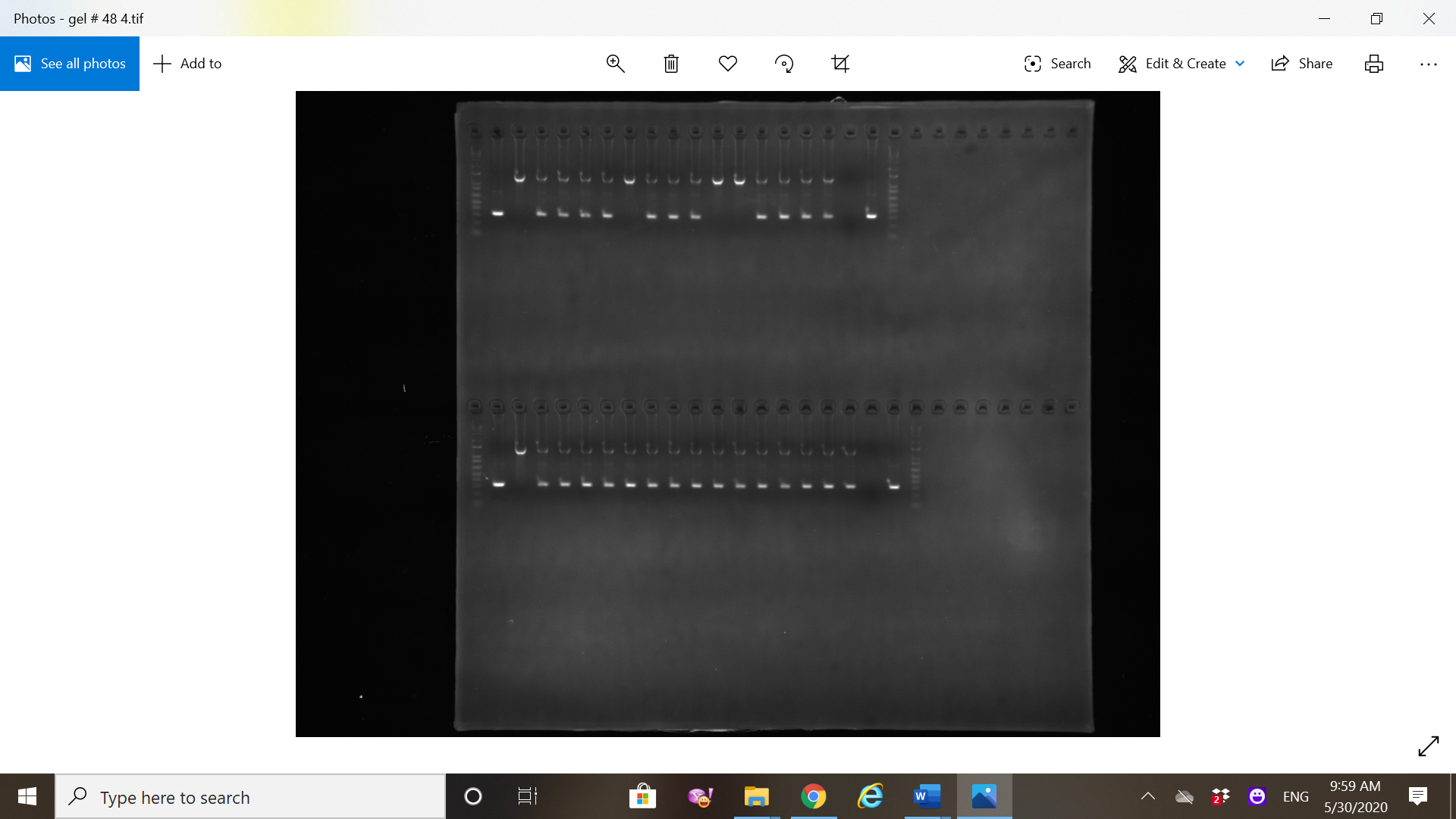


**Supplementary Figure S1**. Original image of gel. Results of PCR detection in Figure 4 is shown. Red box represents the cropped area. Example of PCR analyses of the F_1_ Kansas random generation of transgenic and non-transgenic (full-sibling) channel catfish, *Ictalurus punctatus*, total DNA. Analysis of channel catfish growth hormone (ccGH) cDNA; 332 bp. PCR products visualized with a Molecular Imager Gel Doc XR+ System using Image Lab Software (Bio-Rad Laboratories, Inc, Hercules, CA)^69^. (Photograph by Nermeen Y. Abass).
